# Medical Management of Hemorrhagic Bowel Syndrome in a Beef Bull

**DOI:** 10.1155/2019/9209705

**Published:** 2019-11-03

**Authors:** Joe S. Smith, Xueying Zhou, Paul T. Merkatoris, Cassandra A. Klostermann, Ryan M. Breuer

**Affiliations:** ^1^Veterinary Diagnostic and Production Animal Medicine, Iowa State University, Ames, IA, USA; ^2^Lloyd Veterinary Medical Center, Iowa State University, Ames, IA, USA

## Abstract

A two and a half-year old Simmental bull was presented to Iowa State University's Food Animal and Camelid Hospital for anorexia and lethargy of several days. *Clostridium perfringens* type A was identified via fecal culture and toxin genotyping. Hemorrhagic bowel syndrome (HBS) was diagnosed based on microbiological results along with abdominal ultrasonography, complete blood count, and serum biochemistry. Aggressive multi-modal therapy was employed including intravenously administered fluid therapy, potassium penicillin, lidocaine, flunixin, and pantoprazole among other supportive care. The bull was discharged after 15 days of hospitalization and recovered uneventfully to full function by the next breeding season. Currently all case reports with regard to HBS in beef cattle describe mortality. While the dairy cattle literature demonstrates that HBS has a high mortality rate, and suggests that surgical intervention has a higher prognosis when compared to medical therapy in dairy cattle. Our case would provide support to aggressive medical treatment for HBS in beef cattle.

## 1. Introduction

Hemorrhagic bowel syndrome (HBS), also known as jejunal hemorrhage syndrome (JHS) is an enterotoxemic disease of acute presentation in cattle, predominantly dairy cattle. A relationship between HBS/JHS and *Clostridium perfringens* type A has been established [1]. Affected cattle typically present with profound depression, dehydration, melena, as well as decreased intake decreased milk production, tachycardia, ruminal stasis, abdominal distention, and dark clotted blood in the feces. While multiple cases of this condition are reported for dairy cattle, currently there are few cases reported for beef cattle, all of which demonstrate mortality and lack of response to therapy.

Overall, prognosis with surgical management is only slightly better compared to medical management, with one study reporting that 7/8 medically managed cases died or were euthanized, and 9/13 surgically managed cases having a similar outcome [1]. The three options for surgical management of HBS/JHS include manual clot dissolution with massage, clot removal via enterotomy, and resection and anastomosis of affected intestines. A recent report cited improved survival of surgical cases that utilized manual clot dissolution (4/4) compared to enterotomy (2/6) or resection and anastomosis (1/3) [2]. Several reports note that early surgical intervention can be effective, and factors associated with poor prognosis include puncture of the bowel during manipulation and the involvement of multiple sites within the jejunum and ileum [3]. While descriptions of HBS in the literature are predominantly dairy cattle, there are several cases in beef cattle reported in the literature. These cases range from acute presentation of sudden death [4] to unsuccessful treatment via medical management and surgical clot manipulation [2]. There are currently no reports in the literature of successful treatment of HBS in beef cattle.

Hemorrhagic bowel syndrome is an incompletely understood enteric infection in cattle that, to the authors' knowledge has not been reported to have been successfully treated via medical therapy alone in beef cattle. This report highlights successful non-surgical treatment of hemorrhagic bowel syndrome in a beef bull, utilizing traditional bovine therapies as well as comparative therapies from the large animal literature. Our findings suggest that aggressive initial medical therapy may lead to an additional option to surgery for successful outcomes in cases of HBS in beef cattle.

## 2. Case Description

A 2.5 year old Simmental bull weighing 767 kg was admitted to Iowa State University FACH for decreased appetite and libido beginning three days prior to presentation. The bull had been regularly vaccinated with multivalent clostridial and respiratory disease vaccinations. The bull's diet was composed of a total mix ration (TMR), shell corn as well as pasture grazing. A month prior to presentation, the bull was caught on a gate, and meloxicam (1 mg/kg PO, q 24 × 3) was administered for mild swelling noted in the distal right pelvic limb. Three days prior to presentation to the FACH, the bull was noted to be off feed, and was administered oxytetracycline (20 mg/kg, IM, once), flunixin meglumine (2.2 mg/kg, IV, once), Vitamin B12 (5 mg/kg, IM, once), and thiamine (10 mg/kg, IM, once) by the referring veterinarian. The bull was examined by the referring veterinarian one day prior to presentation to the FACH; the complete blood count (CBC) findings were unremarkable, the serum chemistry revealed hypoproteinemia due to hypoalbuminemia and increased urea nitrogen (BUN) and strongyle-type eggs were identified on fecal flotation.

**Figure 1:**
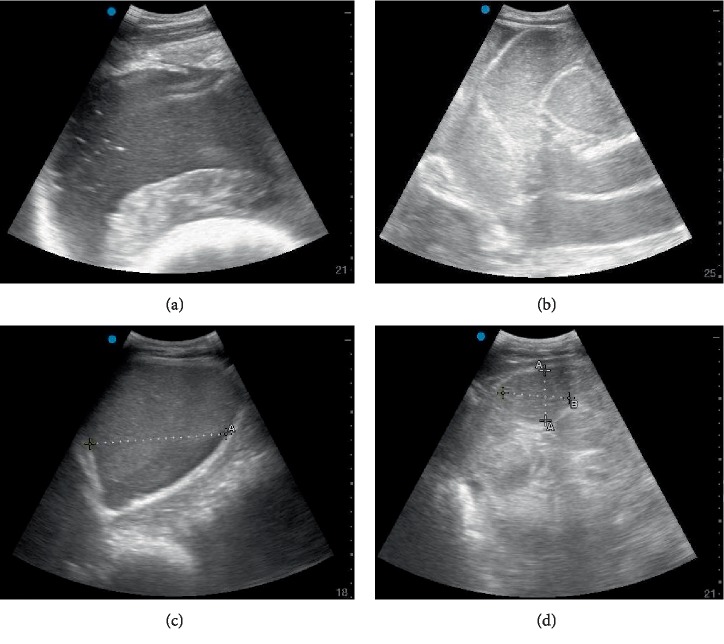
Abdominal Ultrasound. Initial presentation ultrasound—hypoechoic intraluminal fluid of the small intestine in the right caudal ventral abdomen. Screen depth = 21 cm (a). Initial presentation ultrasound—mildly dilated loops of small intestines with hypomotility. Screen depth = 25 cm (b). Day 3 of hospitalization ultrasound—dilated small intestines (range: 4–8 cm in diameter) of the right lateral abdomen, thickening of small intestinal wall. Screen depth = 18 cm (c). Day 6 of hospitalization ultrasound—normal small intestinal luminal diameter (4–5 cm in diameter) with regular peristaltic intervals observed, no luminal distention of small intestine observed. Screen depth = 21 cm (d).

On presentation to the FACH, the bull was quiet, alert, and responsive. The rectal temperature was 38.3°C, heart rate was 100 beats per minute with no murmurs or arrhythmias auscultated, and respiratory rate was 40 breaths per minute with no abnormal lung sounds auscultated. There was one weak rumen contraction per minute and no abnormalities on percussion of the left and right abdomen. The bull was negative to withers pinch and bar (grunt) tests for cranial abdominal pain. Rectal exam revealed melena and clotted blood, and no abnormalities were palpated. The remainder of the physical exam appeared to be unremarkable. Ultrasound of the right caudal ventral abdomen was conducted with a 2–5 MHz curvilinear probe (SonoSite Edge II Ultrasound System, Fujifilm) and revealed hypoechoic intraluminal fluid with loops of intestines that were mildly distended with hypomotile appearance (Figures 1(a) and 1(b)). Liver and gall bladder appeared to be normal. Ultrasound of the ventral left thorax revealed infrequent contractions of the reticulum (1 per 3 minutes; normal: 3 biphasic contractions per 3 minutes [5]).

The hematology results revealed a neutrophilia (7.53; reference range 0.6–4.0 × 10^3^/*µ*L), lymphocytopenia (1.16; reference range 2.5–7.5 × 10^3^/*µ*L), increased MCHC (38.7; reference range 30–36 gm/dL), and the plasma protein to fibrinogen ratio was 10.8. The serum chemistry indicated hypokalemia (3.5; reference range 3.7–5.3 mEq/dL), hypochloremia (81; reference range 94–109 mEq/dL), hypocalcemia (7.7; reference range 8.2–10.1 mg/dL), metabolic alkalosis with a bicarbonate of 43.0 (reference range 19.5–30.3 mEq/dL), azotemia with a BUN of 46 (reference range 7–32 mg/dL) and creatinine of 4.4 (reference range 0.7–1.9 mg/dL), hypoalbuminemia (2.4; reference range 3.2–3.9 mg/dL), hypoproteinemia (6.1; reference range 6.7–8.7 gm/dL), elevated AST (172; reference range 68–156 IU/dL), and hyperbilirubinemia (0.35; reference range <0.1–0.18 mg/dL). Selected hematology and serum biochemistry values are displayed in Table 1. The enteric fecal PCR was negative for Bovine Coronavirus, Rotavirus Group A, *E. coli* (K99+), *Cryptosporidium parvum*, and *Salmonella* spp.

Differential diagnoses included abomasal ulceration, HBS, and other causes of intestinal obstruction (intussusception, intestinal incarceration, volvulus of the mesenteric root). The client was offered laparotomy or medical management, and elected for medical management. Therapy for the bull included polyionic intravenous (IV) fluid therapy (19L 0.9% NaCl, with 40 g KCl and 500 ml 23% calcium borogluconate added), given at a maintenance rate of 1.5 L/hr, and pantoprazole (1 mg/kg IV, q 25 h; Protonix, Pfizer). A single dose of Vitamin K_1_ (0.5 mg/kg, IV; Vetone) which was given at the request of the referring veterinarian.

The second day of hospitalization, the vital parameters were within normal limits (*T* = 38.6°C, *P* = 72 bpm, *R* = 20 br/min), but the bull remained lethargic and anorexic. Urinalysis revealed a specific gravity of 1.014. Chloride and potassium were moderately decreased. Medical management was continued as previously prescribed (IV fluids and pantoprazole), with the addition of thiamine (20 mg/kg IV, once; followed by 10 mg/kg IV, q 12 hr; Vetone).

On the third day, the bull's attitude and appetite were not improved, and no significant passage of manure was noted. Rectal exam revealed distended loops of intestines in the abdomen, feces dark red in color and a gelatinous blood clot was noted in the manure. CBC showed moderate normocytic anemia, and a neutropenia with left shift. Plasma protein to fibrinogen ratio was calculated to be 8.0 (indicating inflammation). Serum biochemistry demonstrated hypochloremia, metabolic alkalosis and azotemia (Table 1). Abdominal ultrasound revealed dilated intestines (4–8 cm) with echoic material consistent with fibrin occasionally seen inside the lumen (Figure 1(c)). A brief thoracic ultrasound, performed as previously described [6], revealed comet tails in lung tissue, indicating pulmonary surface deficits of potential consolidations. A rumen fluid sample, obtained by passage of an orogastric tube, was dark yellow in color with a normal pH (6.5). Transfaunation was performed using 5 L of rumen fluid obtained from a healthy dairy cow. Medical management was continued as previously prescribed (IV fluids, pantoprazole, and thiamine) with the addition of penicillin G potassium (22,000 IU/kg, IV q 6 h; Pfizerpen, Pfizer) and a constant rate infusion (CRI) of lidocaine (0.05 mg/kg/min IV; Xylocaine, Fresenius Kabi). At this time fecal culture was positive for *Clostridium perfringens*.

On the fourth day, improvement was seen by presence of a 15 × 10 cm deposit of loose manure in the stall and resolution of proteinuria. Rectal exam revealed dilated loops of intestines and dark red feces that were subjectively less viscous than previously observed. Serum biochemistry showed that electrolytes disorders were almost completely corrected (Table 1), with improving azotemia, hypoalbuminemia and hypoproteinemia. Abdominal ultrasound showed mildly dilated intestines (2–4 cm) with normal motility. On auscultation, rumen contractions were stronger than previous findings. The IV fluid therapy was continued with a polyionic fluid at a maintenance rate of 1.5 L/h. Medical management was continued as previously prescribed (pantoprazole, thiamine, lidocaine, and penicillin G potassium), with the addition of flunixin meglumine 1.1 mg/kg, IV, once (Prevail, Vetone).

On the fifth day, the bull remained lethargic but his appetite had increased, having eaten 2 flakes of alfalfa hay. CBC improvements in fibrinogen and hematocrit were observed. Medical management was continued as previously prescribed (IV fluids, pantoprazole, thiamine, lidocaine, and penicillin G potassium), and flunixin meglumine frequency was increased (1.1 mg/kg IV, q 12 hr). Genotyping of the *Clostridium perfringens* cultured yielded a type A with the positive presence of alpha toxin.

On the sixth day, the patient was less lethargic with continued intake of 2 flakes of alfalfa hay. Serum biochemistry was within normal limits. Medical management was continued as previously prescribed (pantoprazole, thiamine, lidocaine, penicillin G potassium, and flunixin meglumine), with the addition of administration of an adsorbant (kaolin and pectin, 4 liters, PO, once).

On the seventh day, his temperature was severely elevated (41.2°C) partially attributed to increased ambient temperature (due to an air conditioning failure in the FACH and 32.1°C indoor ambient temperature), which subsequently decreased to 39.1°C after alcohol-evaporative cooling and fan placement. CBC showed an increasing, but still low hematocrit and mildly increased fibrinogen. Serum biochemistry results continued to be within normal limits (Table 1). A repeat abdominal ultrasound revealed frequent peristalsis, without any signs of distention (Figure 1(d)). No free abdominal fluid was noted. Rumen contractions were weak and infrequent. The liver appeared normal with a mildly distended gall bladder and normal vena cava. In addition, gastrointestinal bleeding was assumed discontinued or significantly decreased as evidenced by a negative fecal occult blood test. The lidocaine CRI was discontinued on this day.

On the eighth day, IV fluid therapy, penicillin G potassium, flunixin meglumine, and thiamine were discontinued. Antibiotic therapy was continued with ampicillin trihydrate (10 mg/kg IM, BID; Polyflex, Zoetis) and anti-inflammatory therapy was transitioned to meloxicam (1 mg/kg PO, q 24 hr; Generic).

The last CBC, performed on 11^th^ day, showed an increased hematocrit and a normal fibrinogen concentration. The appetite and attitude of the bull were improved on this day with pacing observed in the stall and a greater amount of hay consumed. On the 15^th^ day, the bull demonstrated a normal appetite as well as fecal production and was discharged. Follow up was achieved via phone or email correspondence with the client and referring veterinarian at 15, 30, 60, and 181 days after discharge. The bull recovered fully, performed well, and demonstrated satisfactory semen quality prior to the fall and spring breeding seasons.

## 3. Discussion

This report describes the presentation of hemorrhagic bowel syndrome in a beef bull, confirmed by abdominal ultrasound, blood clots in the manure, and the culture of *C. perfringens*, subsequently genotyped to type A. This bull responded well to medical management of intravenous fluid therapy, parenteral antibiotics, nonsteroidal anti-inflammatories, lidocaine CRI and pantoprazole. Our findings suggest that aggressive multimodal medical therapy may be key to successful medical treatment of this condition in beef cattle.

The current use of intravenous lidocaine by constant rate infusion in large animal medicine is primarily an equine treatment modality for postoperative ileus. In an equine model of ischemia and reperfusion, lidocaine was found to increase the frequency of contraction of jejunal segments in the control and experimental group both in vivo and in vitro [7, 8]. It is debated whether intravenous lidocaine improves intestinal motility by anti-inflammatory or prokinetic effects [9]. In a study of 126 horses undergoing colic surgery, there was a reduction in the development of postoperative ileus in horses that received lidocaine, and this group was 3.3 times more likely to survive to discharge than those that did not receive lidocaine [10]. It is unknown whether lidocaine had a prokinetic effect in this case, however it was well tolerated by the bull. Due to decreased plasma protein levels in this case and increased sensitivity of ruminants to lidocaine, this bull was not administered a loading dose prior to maintaining the infusion. Lidocaine toxicity in ruminant species is primarily described as neurotoxicity. The sequence of intoxication in sheep is reported as convulsion, hypotension, respiratory arrest, and finally, circulatory collapse [11]. One previous study reports the use of lidocaine intravenously to cattle with HBS, but in that study all cattle treated medically died [1]. While not commonly considered, lidocaine may possess antimicrobial properties [12, 13]. However, other studies have suggested that in humans lidocaine may attenuate the immune response by mechanisms such as suppression of oxygen metabolites and leukocyte chemotaxis inhibition [14]. Lidocaine may have a more prominent role in the future treatment of cattle with HBS.

Currently there are no studies evaluating the use of pantoprazole in cattle. Pantoprazole functions as a proton pump inhibitor which increases gastric pH via bonding with the hydrogen-potassium ATP-ase pump on the secretory surface of gastric parietal cells. It is routinely used in canine and feline practice, and its use has been described for foals [15]. In alpacas [16], pantoprazole has been shown to increase third compartment pH when given at a dose of 1 mg/kg intravenously or 2 mg/kg subcutaneously. Among other ruminant species there are cases reporting the use of pantoprazole in a goat and a yak [6, 17]. Reported complications associated with pantoprazole in people include hyponatremia in hospitalized patients as well as neutropenia and thrombocytopenia with long-term use [18, 19]. The bull in this case did not display neutropenia, thrombocytopenia, or hyponatremia after commencement of pantoprazole therapy. Based on this case and other ruminant cases, pantoprazole may be a safe short-term therapy for protection of the gastrointestinal tract. The safe treatment duration of pantoprazole in cattle is unknown at this time, as increasing the abomasal pH for extended periods of time may lead to the growth of enteropathogenic bacteria. Multiple drugs in this case report, including pantoprazole, were used in an extralabel fashion. Practitioners should be aware that due to regulatory differences, extralabel use of pantoprazole may not be permissible in all countries.

Of the veterinary publications that have described HBS in cattle with respect to etiology, the detection of *C. perfringens* type A is present in the majority of cases. In a retrospective case series of dairy cattle with HBS, *C. perfringens* was cultured from the feces of 17/22 cows, and of cultures that were genotyped, 10/10 were identified as *C. perfringens* type A, with 5/10 having the *β*2 toxin gene [1]. The *β*2 toxin is responsible for inflammation of the intestine as well as partial loss of the mucosa, and the Alpha toxin, a Phospholipase C, is thought to be the primary toxin with respect to hemolysis and necrosis [2]. Genotyping in this case was positive for the Alpha toxin and negative for the *β*2 toxin. Similarly typed isolates were collected from 5 animals in a dairy cattle case report, of which 4/5 died [2]. Antibiotics administered to cattle with HBS in previous reports include procaine penicillin G (22,000 U/kg, IM) [1, 3], ceftiofur sodium (2.2 mg/kg, IM or IV) [1], cefazolin (dose unknown, IV) [20], oxytetracycline (10 mg/kg, IV) [1, 2], or trimethoprim sulfadoxine (16 mg/kg, IV) [2]. The bull in this case report was initially treated with IV penicillin G potassium, and then transitioned to IM ampicillin when clinical signs improved and the IV catheter was removed. At the time of this publication, this is the first report to use penicillin G potassium (22,000 IU/kg IV every 6 hours) for the treatment of HBS, potentially indicating that aggressive multimodal therapy combined with intravenous antimicrobial therapy may be beneficial for HBS.

The present report describes a novel medical management plan for the treatment of HBS in a beef bull, including the use of intravenous fluids, penicillin G potassium, lidocaine, and pantoprazole. Further, this report provides evidence that pantoprazole may be safely tolerated by beef bulls. Future research is necessary to establish safe protocols for the use of lidocaine and pantoprazole in cattle, as well as to determine withdraw recommendations for the use of these drugs in cattle.

## Figures and Tables

**Table 1:** Select hematology and serum biochemistry values from the Simmental bull.

Hematology (CBC) and serum biochemistry parameters	Normal range	Day of hospitalization							
		1	2	3	4	5	6	7	11
Hematocrit	24–46%	29	—	**19.7**	—	**20.4**	—	**21**	**22.1**
White blood cell count	4.0–12.0 × 10^3^/*µ*L	8.96	—	**3.33**	—	**2.93**	—	**1.84**	5.63
Neutrophil count	0.6–4.0 × 10^3^/*µ*L	**7.53**	—	2.33	—	1.7	—	1.44	2.87
Plasma protein	6.9–7.7 gm/dL	**6.5**	—	**6.4**	—	**5.8**	—	**5.1**	**5.7**
Fibrinogen	100–500 mg/dL	**600**	—	**800**	—	**600**	—	**600**	400
Sodium	133–147 mEq/L	135	136	139	143	—	139	137	—
Potassium	3.7–5.3 mEq/L	**3.5**	**3.0**	3.7	4.5	—	4.2	4.8	—
Chloride	94–109 mEq/L	**81**	**90**	**92**	111	—	107	106	—
Bicarbonate	19.5–30.3 mEq/L	**43**	**40**	**37**	23	—	20	25	—
Blood urea nitrogen	7–32 mg/dL	**46**	32	30	25	—	21	15	—
Creatinine	0.7–1.9 mg/dL	**4.4**	—	**3.1**	**2.3**	—	—	1.8	—

Note: Normal ranges as defined by the Iowa State University Veterinary Clinical Pathology Laboratory. Bold values indicate a deviation from the normal range.

## Abbreviations

FACH: Food animal and camelid hospital
HBS: Hemorrhagic bowel syndrome
JHS: Jejunal hemorrhage syndrome
TMR: Total mixed ration.
